# Mediterranean diet in preeclampsia prevention: mechanisms of action and clinical evidence

**DOI:** 10.3389/fnut.2025.1626022

**Published:** 2025-10-02

**Authors:** Dimitris Baroutis, Alexander Papadopoulos, Aikaterini-Gavriela Giannakaki, Eleni Katsianou, Panagiotis Antsaklis, Marianna Theodora, George Daskalakis, Thomas Kotsis

**Affiliations:** ^1^1st Department of Obstetrics and Gynecology, Alexandra Hospital, National and Kapodistrian University of Athens, Athens, Greece; ^2^Aretaieio Hospital, National and Kapodistrian University of Athens, Athens, Greece

**Keywords:** Mediterranean diet, preeclampsia, hypertensive disorders of pregnancy, inflammation, oxidative stress, angiogenic factors, pregnancy outcomes, maternal nutrition

## Abstract

**Introduction:**

Preeclampsia, affecting 2–5% of pregnancies globally, represents a significant challenge in maternal-fetal medicine with substantial morbidity and mortality. Anti-inflammatory dietary patterns, particularly the Mediterranean diet, may ameliorate preeclampsia risk through modulation of inflammatory mediators, oxidative stress, and vascular function. Current findings regarding diet and preeclampsia prevention exhibit considerable variability. We conducted a comprehensive review to evaluate the association between the Mediterranean diet and other anti-inflammatory dietary patterns with preeclampsia risk.

**Methods:**

We performed a systematic literature search across multiple databases including PubMed/MEDLINE, Embase, Google Scholar, ScienceDirect, Scopus, and Web of Science from September 2024 to March 2025. Studies examining Mediterranean Diet or related anti-inflammatory dietary patterns in relation to preeclampsia prevention were included. We extracted data on study design, population characteristics, intervention details, and outcomes related to preeclampsia or hypertensive disorders of pregnancy.

**Results:**

Our analysis included 12 studies (5 observational studies, 7 randomized controlled trials) with diverse geographical representation. Observational studies demonstrated significant protective associations between higher Mediterranean diet adherence and reduced preeclampsia risk, with effect sizes ranging from 22 to 69% risk reduction. Among the RCTs, two reported statistically significant reductions in hypertension/preeclampsia, while four showed protective trends that did not reach statistical significance. Notable dietary components included extra virgin olive oil and nuts, with variable intervention timing across studies.

**Discussion:**

The findings suggest that Mediterranean diet adherence may reduce preeclampsia risk through multiple complementary pathways: reducing inflammatory markers, alleviating oxidative stress, enhancing endothelial function, balancing angiogenic factors, and improving metabolic parameters. Despite methodological limitations, the Mediterranean diet represents a promising non-pharmacological approach to preeclampsia prevention. Future research should address methodological heterogeneity, expand studies to diverse populations, and elucidate optimal timing and specific components of dietary interventions.

## 1 Introduction

Preeclampsia represents one of the most significant challenges in maternal-fetal medicine, affecting 2–5% of pregnancies globally and constituting a major cause of maternal and neonatal morbidity and mortality (1, 2). This pregnancy-specific hypertensive disorder is characterized by new-onset hypertension after 20 weeks of gestation accompanied by proteinuria and/or maternal organ dysfunction (3, 4). Despite decades of research and advances in prenatal care, preeclampsia remains a leading cause of maternal mortality, accounting for approximately 46,000 maternal deaths and 500,000 perinatal deaths annually (1). The disease burden is disproportionately borne by women in low- and middle-income countries or those otherwise disadvantaged, highlighting significant health disparities in maternal care worldwide (5, 6). The global incidence of preeclampsia has remained relatively stable over time, though regional variations exist. Incidence rates range from as low as 1% in some European countries to over 7% in certain developing regions (1, 2). This geographic variability reflects differences in risk factors, healthcare access, diagnostic criteria, and possibly dietary patterns across populations (1, 5). In the United States, preeclampsia affects approximately 3–5% of pregnancies and is responsible for 15% of maternal deaths and 25% of medically indicated preterm deliveries (7, 8). The pathophysiology of preeclampsia involves complex interactions between placental and maternal factors, making it a two-stage disorder. The first stage involves abnormal placentation during early pregnancy, while the second stage encompasses the maternal systemic response that leads to the clinical syndrome (9, 10). The primary pathogenic event is believed to be inadequate trophoblastic invasion of maternal spiral arteries during placentation. In normal pregnancy, trophoblasts invade and remodel maternal spiral arteries, transforming them from high-resistance, low-capacity vessels into low-resistance, high-capacity vessels capable of meeting the increasing demands of the growing fetus. In preeclampsia, this remodeling is impaired, resulting in narrow, high-resistance vessels (9, 10). This inadequate placentation leads to placental hypoxia, ischemia, oxidative stress, and the release of various factors into the maternal circulation, including anti-angiogenic factors, inflammatory cytokines, syncytiotrophoblast microparticles, and cell-free fetal DNA (9–11). Among these, the imbalance between pro-angiogenic factors (such as placental growth factor, PlGF) and anti-angiogenic factors (such as soluble fms-like tyrosine kinase-1, sFlt-1, and soluble endoglin, sEng) plays a crucial role. Elevated levels of sFlt-1 sequester circulating PlGF and vascular endothelial growth factor (VEGF), preventing their interaction with endothelial cell receptors and leading to endothelial dysfunction (9–11). This endothelial dysfunction represents the cornerstone of the maternal syndrome and affects multiple organ systems. The resulting systemic inflammatory response, increased vascular reactivity, and activation of the coagulation cascade contribute to the clinical manifestations of preeclampsia (11). Recent research has also implicated additional factors in preeclampsia pathogenesis, including immune maladaptation, genetic predisposition, excessive complement activation, and alterations in the renin-angiotensin-aldosterone system (9–11).

Preeclampsia has been traditionally classified based on the timing of onset, with important prognostic and pathophysiological implications. Early-onset preeclampsia (developing before 34 weeks’ gestation) is more often associated with inadequate placentation, severe maternal and fetal complications, and a hemodynamic profile of low cardiac output and high peripheral vascular resistance (12). This subtype is more likely to be associated with fetal growth restriction, reflecting the significant placental involvement in its pathogenesis. In contrast, late-onset preeclampsia (at or after 34 weeks’ gestation), which accounts for at least 70% of cases, usually presents with normal or even increased birth weight, potentially increased cardiac output, and variable peripheral vascular resistance [either decreased (12) or increased (13)]. While much of the literature focuses on preterm preeclampsia, which is associated with a substantially higher risk of maternal and fetal complications than term preeclampsia, the latter accounts for approximately two-thirds of cases and makes a substantial contribution to overall preeclampsia-related morbidity (7, 8). More recently, preeclampsia has been subclassified based on the presence or absence of severe features, which include severe hypertension, severe proteinuria, thrombocytopenia, impaired liver function, renal insufficiency, pulmonary edema, new-onset cerebral or visual disturbances, and uteroplacental insufficiency (3, 4). This classification has important implications for management and prognosis.

Multiple risk factors for preeclampsia have been identified, often categorized as maternal, paternal, and placental factors. Maternal biologic and social risk factors include certain demographic characteristics (e.g., membership in a minority racial or ethnic group, age extremes), a history of medical or obstetrical disorders (e.g., chronic hypertension, pre-existing diabetes, previous preeclampsia, first pregnancy), certain characteristics of the current pregnancy (e.g., multifetal gestation, conception by means of assisted reproductive technology), physiological abnormalities (e.g., increased blood pressure, obesity), abnormal results of laboratory tests (e.g., severe anemia), and ultrasonographic abnormalities (e.g., an abnormal uterine-artery pulsatility index, measured by Doppler ultrasonography) (3, 11). Risk assessment for preeclampsia is crucial for implementing preventive strategies in high-risk populations. Traditional screening involves assessment of clinical risk factors early in pregnancy, treating them independently and summarizing them without indicating the level of risk or as a count of factors (1). This approach is simple but has low detection rates for both preterm preeclampsia (approximately 40%) and term preeclampsia (approximately 35%), with a positive screening rate of approximately 10% (3). More sophisticated multivariable models have been developed with high detection rates when used at 11 to 13 weeks’ gestation for preterm preeclampsia and at 35 to 36 weeks’ gestation for term preeclampsia. The competing-risks model of the Fetal Medicine Foundation (FMF), which is supported by substantial evidence, incorporates maternal characteristics (including ethnic/racial background and body mass index), blood pressure, uterine-artery pulsatility index, and angiogenic markers (5). This model can identify approximately 90% of women at 11 to 13 weeks’ gestation in whom early preeclampsia will develop and approximately 75% of those who will develop preterm preeclampsia, with a positive screening rate of 10% (3).

The prevention of preeclampsia remains a top priority in maternal healthcare, as the only definitive treatment is delivery of the placenta, which has been shown to initiate the resolution of preeclampsia once it has developed. Current preventive strategies focus on addressing the pathophysiological mechanisms involved in preeclampsia development, including angiogenic imbalance, endothelial activation, oxidative stress, inflammation, and vasoconstriction (11). Several pharmacological interventions have been studied extensively, with low-dose aspirin being the most widely recommended for high-risk women (14, 15). A meta-analysis of 60 trials involving 36,716 women at increased risk for preeclampsia demonstrated that aspirin (50 to 162 mg per day, usually ≤75 mg per day) reduces the risk of preeclampsia in a dose-dependent manner (relative risk, 0.82; 95% CI, 0.77 to 0.88), along with lowering the rates of serious maternal complications, preterm birth, delivery of small-for-gestational-age infants, and fetal or newborn death (10). In the landmark ASPRE (Combined Multimarker Screening and Randomized Patient Treatment with Aspirin for Evidence-Based Preeclampsia Prevention) trial, aspirin (150 mg per day) administered from 11 to 13 weeks’ gestation until 36 weeks’ gestation reduced the risk of preterm preeclampsia by more than 60% (odds ratio, 0.38; 95% CI, 0.20 to 0.74) (14). A subsequent meta-analysis confirmed that aspirin is beneficial in preventing preterm preeclampsia (relative risk, 0.62; 95% CI, 0.45 to 0.87) but not term disease, provided that treatment is initiated by 16 weeks’ gestation and at a dose of at least 100 mg per day (15). Calcium supplementation is another effective preventive strategy, particularly for women with low dietary calcium intake. A meta-analysis of 30 trials involving 20,445 women showed that calcium supplementation during pregnancy reduces the risk of preeclampsia (relative risk, 0.49; 95% CI, 0.39 to 0.61), with efficacy observed primarily in women with low baseline calcium intake (<900 mg per day) (16). Other pharmacological interventions have been investigated, including pravastatin, folic acid, low-molecular-weight heparin, and metformin, but evidence supporting their routine use for preeclampsia prevention remains limited. Pravastatin, which has lipid-lowering, anti-inflammatory, and pro-angiogenic properties, has shown promise in preliminary studies but requires further investigation in larger trials. Similarly, while some observational studies have suggested a protective effect of folic acid supplementation beyond the recommended dose for neural tube defect prevention, randomized trials have not consistently supported this finding (15, 16).

Non-pharmacological approaches to preeclampsia prevention have also been explored, with varying levels of evidence. Exercise has been shown to reduce the risk of preeclampsia (odds ratio, 0.59; 95% CI, 0.37 to 0.90) without adverse fetal effects (9). To achieve these benefits, women must undertake at least 140 min per week of moderate-intensity exercise. The mechanisms by which exercise reduces preeclampsia risk may include improved placental perfusion, enhanced antioxidant defenses, reduced inflammation, and improved endothelial function (9). Weight management before and during pregnancy represents another potential preventive strategy, given the strong association between obesity and preeclampsia risk. However, evidence for the effectiveness of weight loss interventions initiated during pregnancy is limited and potentially concerning, as excessive weight loss may adversely affect fetal growth. Pre-pregnancy weight optimization appears to be a more promising approach (17). Timed delivery is another strategy for preventing complications of preeclampsia, with labor induction at 39 weeks 0 days to 39 weeks 4 days of gestation reducing the risks of gestational hypertension and preeclampsia in low-risk nulliparous women (17). Lifestyle modifications, particularly dietary interventions, have gained increasing attention as potential preventive measures for preeclampsia. Diet represents a modifiable risk factor that may influence the pathophysiological mechanisms underlying preeclampsia, including inflammation, oxidative stress, endothelial function, and angiogenic balance (17). Multiple dietary factors have been associated with preeclampsia risk. One systematic review found that higher intake of fruits, vegetables, whole grains, and plant-based foods was associated with lower risk of preeclampsia (17). Conversely, diets high in processed foods, refined sugars, and saturated fats may increase risk. Specific nutrients, including antioxidants, omega-3 fatty acids, vitamin D, and dietary fiber, may also play protective roles (18).

Rather than focusing on individual nutrients, research has increasingly examined dietary patterns, which may better capture the complex interactions among various food components. In this context, the Mediterranean diet has emerged as a promising dietary pattern that may reduce the risk of preeclampsia and other pregnancy complications (18, 19). It is characterized by high consumption of plant-based foods (fruits, vegetables, legumes, nuts, and whole grains), moderate consumption of fish and seafood, limited intake of dairy products and red meat, olive oil as the primary source of fat, and moderate wine consumption (though alcohol is typically avoided during pregnancy) (20, 21). The Mediterranean diet is not merely a diet but rather a lifestyle that encompasses cultural, social, and culinary traditions. It was recognized by UNESCO as an Intangible Cultural Heritage of Humanity in 2010, reflecting its cultural significance beyond its nutritional aspects. The traditional Mediterranean diet varies somewhat by region but maintains core elements that are associated with its health benefits (20, 21). The Mediterranean diet is recognized for its anti-inflammatory, antioxidant, and cardioprotective properties. These beneficial effects are attributed to the synergistic interactions among its components, which provide a rich source of bioactive compounds, including polyphenols, monounsaturated and polyunsaturated fatty acids, fiber, vitamins, and minerals (20, 21). Numerous epidemiological studies and clinical trials have demonstrated the health benefits of the Mediterranean diet across diverse populations. It has been associated with reduced risk of cardiovascular disease, type 2 diabetes, metabolic syndrome, certain cancers, and neurodegenerative diseases (20, 21). The landmark PREDIMED (Prevención con Dieta Mediterránea) trial demonstrated that a Mediterranean diet supplemented with extra-virgin olive oil or nuts reduced the risk of major cardiovascular events by approximately 30% in individuals at high cardiovascular risk (20). In recent years, there has been growing academic interest in its potential to yield beneficial effects for reproductive health and pregnancy-related outcomes (18, 19).

The Mediterranean diet contains several components that may collectively contribute to preeclampsia prevention through mechanisms targeting its pathophysiology (19). Plant-based foods in the Mediterranean diet provide antioxidants including vitamins C and E, carotenoids, and polyphenols that counteract oxidative stress—a central element in preeclampsia pathogenesis contributing to placental dysfunction and endothelial damage (22, 23). These compounds work synergistically, potentially explaining why whole foods appear more beneficial than isolated antioxidant supplements in preeclampsia prevention (21–23). The Mediterranean diet ‘s inclusion of fatty fish supplies omega-3 fatty acids (EPA and DHA) that modulate inflammatory mediators and angiogenic factors, addressing the systemic inflammation and vascular dysregulation characteristic of preeclampsia (24, 25). Extra virgin olive oil (EVOO), a main fat source in the Mediterranean diet, along with olive oil, contains oleocanthal with anti-inflammatory properties and polyphenols that improve endothelial function while favorably affecting lipid profiles and insulin sensitivity (26, 27). Nuts, seeds, and legumes contribute L-arginine (a precursor for nitric oxide critical for vascular tone regulation), magnesium (with vascular smooth muscle relaxant properties), and potassium (for blood pressure regulation)—all addressing mechanisms implicated in preeclampsia (20, 21). Additionally, the high fiber content from fruits, vegetables, whole grains, and legumes promotes beneficial gut microbiota, potentially preventing bacterial translocation and endotoxemia that may contribute to preeclampsia-associated inflammation (28, 29). The Mediterranean diet’s protective effects likely operate through multiple complementary pathways: reducing inflammatory markers including C-reactive protein and interleukin-6 (30), alleviating oxidative stress at the maternal-fetal interface (31), enhancing endothelial function through increased nitric oxide bioavailability (32, 33), balancing angiogenic factors by reducing anti-angiogenic sFlt-1 while increasing pro-angiogenic PlGF (34, 35), and improving metabolic parameters relevant to preeclampsia risk (20, 21). These mechanisms directly address the key pathophysiological processes involved in preeclampsia development, suggesting biological plausibility for the Mediterranean diet’s potential protective effects.

Research on Mediterranean diet and preeclampsia has been limited, with earlier studies focusing on specific nutrients rather than overall dietary patterns (36, 37). Evidence quality varies considerably, with many observational studies subject to confounding (38, 39). Methodological inconsistencies complicate interpretation, as different scoring systems for Mediterranean diet adherence make direct comparisons challenging. Most research has been conducted primarily in White populations, despite preeclampsia disproportionately affecting racial and ethnic minority groups (5, 6). The optimal timing and duration of Mediterranean diet interventions remain unclear. While earlier implementation may offer greater benefits, more research is needed to identify critical periods when dietary modifications most effectively reduce preeclampsia risk (17). Understanding Mediterranean diet’s protective mechanisms is essential for developing evidence-based recommendations. If proven effective, Mediterranean diet adherence could represent a low-cost, non-pharmacological approach to reducing preeclampsia risk, particularly where medical interventions are limited (40, 41). This review synthesizes current evidence regarding Mediterranean diet in preeclampsia prevention, examining both mechanistic pathways and clinical outcomes. By analyzing available literature, we aim to identify knowledge gaps and guide future research in maternal nutrition and pregnancy outcomes.

## 2 Materials and methods

This narrative review was conducted to synthesize and analyze the existing literature on the Mediterranean diet and its role in preeclampsia prevention. While we followed a structured approach to literature search and selection, this review was not conducted according to PRISMA guidelines, as it is not a systematic review or meta-analysis.

### 2.1 Search strategy

A comprehensive literature search was performed using multiple databases and online platforms including the following: PubMed/MEDLINE, Embase, Google Scholar, ScienceDirect, Scopus, and Web of Science (Clarivate). The search was conducted from September 2024 to March 2025. The following search algorithm was used:

(Preeclampsia OR Pre-eclampsia OR toxaemia OR toxemia OR eclampsia) AND (“Mediterranean diet”)

Additionally, the ‘snowball literature searching method’ was employed to identify additional relevant sources from the reference lists of selected articles.

### 2.2 Study selection

The selection of included studies was carried out based on their relevance to the subject in terms of their title and abstract, followed by a full-text examination. The inclusion criteria were:

1. Studies examining the Mediterranean diet as a whole dietary pattern in relation to preeclampsia prevention or risk reduction.
2. Studies focusing on pregnant women or women of reproductive age.
3. Published original research studies, including randomized controlled trials, cohort studies, case–control studies, and cross-sectional studies.
4. Studies including methods for evaluating adherence to the Mediterranean diet.

Exclusion criteria were:

1. Studies focusing only on specific ingredients, vitamins, or trace elements rather than the Mediterranean diet or Mediterranean based diets.
2. Studies not addressing preeclampsia or hypertensive disorders of pregnancy as outcomes.
3. Studies focusing only on other pregnancy complications without mention of preeclampsia.

Literature reviews, systematic reviews, and case reports (though these were examined for potential primary sources).

There was no time limit applied, and only English language publications were considered.

### 2.3 Data extraction and synthesis

Two reviewers (D.B. and A.P.) independently extracted data from the selected studies using a standardized form. The extracted information included the following: first author, publication year, country, study design, sample size and characteristics, method of evaluating Mediterranean diet adherence, duration of study and follow-up period, main results, and confounding factors considered.

Our review evaluated the association between adherence to the Mediterranean diet before and/or during pregnancy and the risk of developing preeclampsia. We examined both the direct associations with preeclampsia as well as effects on related pathophysiological mechanisms.

### 2.4 Quality assessment

While a formal quality assessment of included studies was not conducted due to the narrative nature of this review, we critically evaluated each study’s methodology, sample size, and potential biases during our analysis and interpretation of results. Our evaluation considered the following aspects:

- Study design: we assessed whether the design (randomized controlled trial, cohort, case–control, cross-sectional) was appropriate for addressing the research question.
- Sample size
- Mediterranean diet assessment methods: we evaluated the tools used to measure diet adherence, including the number of food items assessed and whether the tools were validated.
- Outcome measures
- Confounding factors
- Reporting of results: we assessed the clarity and completeness of the reported findings.

## 3 Results

### 3.1 Overview of included studies

The literature search yielded 12 studies that met our inclusion criteria, comprising both observational studies (5 prospective cohort studies) and interventional studies (7 randomized controlled trials). The studies were conducted across diverse geographical regions, including Australia, Spain, Greece, the United Kingdom, China, and the United States. Sample sizes ranged from 82 to 8,507 participants, with studies published between 2015 and 2023. The included studies examined the relationship between Mediterranean diet (MD) adherence and hypertensive disorders of pregnancy (HDP), including preeclampsia and gestational hypertension.

### 3.2 Observational studies

#### 3.2.1 Australian Longitudinal Study on Women’s Health

This population-based prospective cohort study of 3,582 Australian women found a significant association between adherence to a Mediterranean-style dietary pattern before pregnancy and reduced risk of developing hypertensive disorders during pregnancy. Women in the highest quartile of Mediterranean diet adherence had a 42% lower risk of developing HDPs compared to those in the lowest quartile (RR 0.58; 95% CI, 0.42, 0.81). This study provided valuable evidence on the potential protective effects of pre-pregnancy dietary patterns on HDP risk (42).

#### 3.2.2 Mediterranean diet adherence and prematurity study

This smaller observational cohort study (*n* = 82) of women who delivered preterm singletons at ≤34 weeks demonstrated that low Mediterranean diet adherence was associated with higher rates of hypertension/preeclampsia. Specifically, women with low Mediterranean diet adherence had 3.8 times higher odds of developing hypertension/preeclampsia compared to those with high adherence (OR 3.8; 95% CI 1.3–11.4, *p* = 0.019) (43).

#### 3.2.3 Healthy Dietary Patterns Study

This prospective cohort study evaluated 1,887 pregnant women from the Eunice Kennedy Shriver National Institute of Child Health and Human Development Fetal Growth Studies. Researchers assessed Alternate Mediterranean Diet (AMED) scores at three time points during pregnancy (8–13, 16–22, and 24–29 weeks). The Alternate Mediterranean Diet (AMED) score is a modified version of the Mediterranean Diet score (MDS) designed to assess adherence to a Mediterranean-style diet, particularly within populations outside the Mediterranean region, such as those in the United States. It typically ranges from 0 to 9, with higher scores indicating greater adherence (44). Higher AMED scores at 8–13 weeks gestation were significantly associated with a lower risk of preeclampsia (adjusted RR for highest vs. lowest quartile: 0.31 [95% CI: 0.11, 0.87], P-trend = 0.03), suggesting that during periconception and/or early pregnancy adherence to a Mediterranean-style diet may be particularly protective (44).

#### 3.2.4 Boston Birth Cohort

This prospective study included a racially diverse, urban, low-income cohort of 7,770 women. The study utilized a Mediterranean-style diet score (MSDS) derived from food frequency questionnaires administered 24–72 h postpartum. Higher Mediterranean diet adherence was associated with lower preeclampsia odds (highest vs. lowest tertile aOR 0.78, 95% CI 0.64–0.96). Notably, the protective effect was stronger among Black women, highlighting potential racial differences in the benefits of Mediterranean diet adherence (45).

#### 3.2.5 nuMoM2b Study

This large prospective multicenter cohort study included 7,798 nulliparous women with singleton pregnancies across the United States. Using the Alternate Mediterranean Diet (aMed) score based on periconceptional dietary intake, researchers found that high vs. low aMed score was associated with 28% lower odds of preeclampsia (aOR 0.72, 95% CI 0.55–0.93). A dose–response relationship was observed, further strengthening the evidence for the protective effect of Mediterranean diet adherence on preeclampsia risk (46).

### 3.3 Randomized controlled trials

#### 3.3.1 St. Carlos GDM Prevention Study

This randomized controlled trial of 874 normoglycemic pregnant women (440 control, 434 intervention) implemented a Mediterranean diet intervention supplemented with extra virgin olive oil (EVOO, 1 L/week) and pistachios (150 g/week) starting at 8–12 gestational weeks. While the primary outcome was gestational diabetes mellitus (GDM), the study reported a reduction in pregnancy-induced hypertension in the intervention group (11/440cg,7/434ig), although specific numerical results were not detailed in the provided table (47).

#### 3.3.2 ESTEEM trial

This multicenter, clinic-based randomized controlled trial included 1,138 obese pregnant women (BMI > 30 kg/m^2^). The intervention consisted of Mediterranean diet advice supplemented with mixed nuts (30 g/day) and EVOO (0.5 L/week) from 18 gestational weeks until delivery. There was no significant reduction in the prevalence of preeclampsia (aOR 0.76, 95% CI 0.56–1.03) (48).

#### 3.3.3 St Carlos Study

This prospective, universal, interventional study included 932 normoglycemic women before 12 gestational weeks who received Mediterranean diet recommendations with emphasis on EVOO and nuts consumption. Compared to a historical control group, no significant differences were observed in pregnancy-induced hypertension (1.9% vs. 2.3%, RR 1.20, 95% CI 0.42–3.41) or preeclampsia (1.1% vs. 0.8%, RR 0.72, 95% CI 0.11–4.62) (49).

#### 3.3.4 Kang Study

This hospital-based randomized controlled trial of 660 women (332 intervention, 328 control) in China implemented Mediterranean diet advice with recommended walnuts (25–30 g/day) and sunflower seed oil (25–40 mL/day) in normoglycemic women with singleton pregnancies. The intervention showed a trend toward reduction in gestational hypertension (RR 0.71; 95% CI, 0.32–1.57) and preeclampsia (RR 0.62; 95% CI, 0.20–1.87), although these results did not reach statistical significance (50).

#### 3.3.5 Crovetto Study

This hospital-based, 3 parallel-group randomized controlled trial included 1,184 high-risk pregnant women for small-for-gestational-age births (392 Mediterranean diet, 391 mindful-based stress reduction, 401 control). The Mediterranean diet intervention was supplemented with EVOO (2 L/month) and walnuts (15 g/day) from 19 to 23 gestational weeks until 34–36 gestational weeks. The study found a reduced risk of preeclampsia in the Mediterranean diet group (RR 0.61; 95% CI, 0.37–1.01) (51).

#### 3.3.6 Zhao study

This hospital-based controlled trial in China included normoglycemic women with singleton pregnancies, randomizing 280 to intervention and 280 to a control group. The intervention consisted of Mediterranean diet advice with supplemented olive oil (40 mL/day) and pistachios (25–30 g/day), along with recommended walking (30 min/day). The study reported a non-significant reduction in gestational hypertension (RR 0.67; 95% CI, 0.24–1.85) (52).

#### 3.3.7 PREDG study

This randomized controlled trial focused on 169 women with pregestational obesity (BMI ≥ 30 kg/m^2^). The intervention consisted of a group education program on Mediterranean diet (50% carbohydrates, 20% protein, 30% fat) and physical activity. The study reported significantly reduced rates of hypertension/preeclampsia in the intervention group compared to the control group receiving conventional follow-up with standard dietary advice (18.2% vs. 32.5%, *p* = 0.040) (53).

### 3.4 Mediterranean diet components and timing

Across the included studies, various Mediterranean diet components were emphasized in interventions. Extra virgin olive oil and nuts (particularly walnuts and pistachios) were commonly supplemented in intervention groups (47, 48, 50–52). The timing of Mediterranean diet assessment or intervention varied, with some studies focusing on pre-pregnancy dietary patterns (42), others on early pregnancy (8–13 weeks) (44, 47, 50), and some implementing interventions in mid-pregnancy (18–23 weeks) (48, 51).

### 3.5 Summary of findings

Overall, 4 out of 5 observational studies demonstrated statistically significant associations between higher Mediterranean diet adherence and reduced risk of preeclampsia or hypertensive disorders of pregnancy (42, 43, 45, 46). The effect sizes ranged from 22 to 69% risk reduction, with the strongest effects observed in the Healthy Dietary Patterns Study (44), which reported a 69% lower risk of preeclampsia in women with the highest AMED scores (44).

Among the seven randomized controlled trials, two reported statistically significant reductions in hypertension/preeclampsia (47, 53), while four showed trends toward protection that did not reach significance (48, 50–52). One trial reported no effect (49).

Notable differences in study design, population characteristics, timing of intervention, specific Mediterranean diet components, and outcome definitions may explain the variability in results across studies. Nevertheless, the overall trend suggests a potential protective effect of Mediterranean diet adherence against preeclampsia and hypertensive disorders of pregnancy (Table 1).

**Table 1:** The included studies of the review and their key characteristics.

Study	Authors	Year	Country	Study design	Sample size	Population characteristics	Intervention/exposure	Control/comparison	Primary outcomes	Key findings for preeclampsia/HDP	Strengths/limitations
1. Australian Longitudinal Study on Women’s Health	Schoenaker et al. (42)	2015	Australia	Population-based prospective cohort	3,582 women	Women born in 1973–1978, not pregnant at baseline (2003)	Mediterranean-style dietary pattern characterized by vegetables, legumes, nuts, tofu, rice, pasta, rye bread, red wine, and fish	Comparison across quartiles of Mediterranean diet adherence	Hypertensive disorders of pregnancy (HDP) including gestational hypertension and pre-eclampsia	Mediterranean-style dietary pattern associated with reduced risk of HDPs (quartile 4 vs. quartile 1: RR 0.58; 95% CI, 0.42, 0.81)	Strengths: Population-based design, prospective assessment of dietary patterns before pregnancy, long-term follow-up, adjustment for multiple confounders Limitations: Self-reported HDPs, unable to distinguish between subtypes of HDPs, unable to account for dietary changes during pregnancy
2. St. Carlos GDM Prevention Study	Assaf-Balut et al. (47)	2017	Spain	Randomized controlled trial	874 women (440 control, 434 intervention)	Normoglycemic pregnant women at 8–12 gestational weeks	Mediterranean diet supplemented with extra virgin olive oil (EVOO, 1 L/wk) and pistachios (150 g/wk)	Standard dietary advice with limited fat intake; recommended 30 min/d of walking	Incidence of GDM at 24–28 gestational weeks; pregnancy-induced hypertension	Reduced incidence of pregnancy-induced hypertension (specific results not detailed in table)	Strengths: Randomized design, early intervention, provision of key Mediterranean diet components Limitations: Single-center study, limited generalizability
3. Mediterranean Diet Adherence and Prematurity Study	Parlapani et al. (43)	2017	Greece	Prospective, observational cohort	82 women	Women who delivered preterm singletons at ≤34 weeks	Mediterranean diet adherence measured using validated score	Comparison between high vs. low Mediterranean diet adherence (50th centile cutoff)	Intrauterine growth; complications of prematurity	High MDA group had lower rates of hypertension/preeclampsia (OR 3.8; 95% CI 1.3–11.4, *p* = 0.019)	Strengths: First study examining MDA in preterm deliveries Limitations: Small sample size, single-center design, potential recall bias in dietary assessment
4. ESTEEM	Al Wattar et al. (48)	2019	UK	Multicenter, clinic-based, randomized controlled trial	1,138 women (553 vs. 585)	>16 y of age, singleton pregnancy with metabolic risk factors (BMI > 30 kg/m^2^)	MD advice supplemented with mixed nuts (30 g/d) and EVOO (0.5 L/wk); from 18 GW until delivery	Standard dietary advice as per UK national recommendations	GDM, preeclampsia, preterm birth, maternal weight gain, lipids profile	No significant reduction in composite maternal outcome (aOR 0.76, 95% CI 0.56–1.03)	Strengths: Large multicenter trial, diverse population Limitations: Intervention started in mid-pregnancy, self-reported adherence
5. St Carlos Study	García de la Torre et al. (49)	2019	Spain	Prospective, universal, interventional study	932 women	Normoglycemic women before 12 gestational weeks	Mediterranean diet recommendations with emphasis on EVOO and nuts consumption	Comparison with historical control group	GDM rate and adverse maternal-fetal outcomes	No significant differences in pregnancy-induced hypertension (1.9% vs. 2.3%, RR 1.20, 95% CI 0.42–3.41) or preeclampsia (1.1% vs. 0.8%, RR 0.72, 95% CI 0.11–4.62)	Strengths: Implementation in real-world setting, early intervention Limitations: Lack of randomization, historical control group
6. Kang Study	Kang et al. (50)	2019	China	Hospital-based randomized controlled trial	660 women (332 vs. 328)	18–45 y of age, singleton pregnancy, normoglycemic (6 mmol/L)	MD advice with recommended walnuts (25–30 g/d) and sunflower seed oil (25–40 mL/d); PA not mentioned	Standard dietary advice with limited fat and sugar intake; PA not mentioned	GDM, gestational hypertension, preeclampsia, preterm birth, maternal weight gain	Reduction in gestational hypertension (RR 0.71; 95% CI, 0.32–1.57) and preeclampsia (RR 0.62; 95% CI, 0.20–1.87)	Strengths: Randomized design, early intervention starting at 8–12 GW Limitations: Single-center study, self-reported adherence
7. Crovetto Study	Crovetto et al. (51)	2021	Spain	Hospital-based, 3 parallel-group randomized controlled trial	1,221 women (392 vs. 391 vs. 401)	>18 y of age, singleton pregnancy, high-risk for SGA	MD advice supplemented with EVOO (2 L/mo) and walnuts (15 g/d); from 19–23 GW until 34–36 GW	Mindful-based stress reduction arm, control arm receiving usual pregnancy care	Preterm birth, preeclampsia, GDM, maternal weight gain	Reduced risk of preeclampsia (RR 0.61; 95% CI, 0.37–1.01)	Strengths: Multi-arm design, intervention in high-risk population Limitations: Late intervention timing (19–23 GW), limited power for some outcomes
8. Healthy Dietary Patterns Study	Li et al. (44)	2021	USA	Prospective cohort	1,887 pregnant women	Women from the Eunice Kennedy Shriver National Institute of Child Health and Human Development Fetal Growth Studies	Alternate Mediterranean diet (AMED) score assessed at 8–13, 16–22, and 24–29 weeks	Comparison across quartiles of dietary pattern scores	GDM, gestational hypertension, preeclampsia, preterm delivery	Higher AMED score at 8–13 weeks associated with lower risk of preeclampsia (adjusted RR [95% CI], Q4 vs. Q1: 0.31 [0.11, 0.87], P-trend = 0.03)	Strengths: Longitudinal dietary assessment, multiple dietary patterns assessed Limitations: Potential selection bias, self-reported dietary intake
9. Boston Birth Cohort	Minhas et al. (45)	2022	USA	Prospective cohort study	8,507 women (7,770 after exclusions)	Racially diverse, urban, low-income women	Mediterranean-style diet score (MSDS) derived from food frequency questionnaire administered 24–72 h postpartum	Comparison across tertiles of Mediterranean diet adherence	Preeclampsia	Higher Mediterranean diet adherence associated with lower preeclampsia odds (highest vs. lowest tertile aOR 0.78, 95% CI 0.64–0.96); Stronger protective effect in Black women	Strengths: Large, diverse sample, detailed analysis by race Limitations: Cross-sectional dietary assessment, potential recall bias
10. nuMoM2b	Makarem et al. (46)	2022	USA	Prospective multicenter cohort	7,798 women	Nulliparous women with singleton pregnancies	Alternate Mediterranean Diet (aMed) score based on periconceptional dietary intake assessed using food frequency questionnaire	Comparison across tertiles and quintiles of Mediterranean diet adherence	Any adverse pregnancy outcome (APO) defined as preeclampsia/eclampsia, gestational hypertension, gestational diabetes, preterm birth, small-for-gestational-age infant, or stillbirth	High vs. low aMed score associated with 28% lower odds of preeclampsia (aOR 0.72, 95% CI 0.55–0.93); Dose–response relationship observed	Strengths: Geographically diverse US population, prospective design Limitations: Self-reported diet, potential recall bias
11. Zhao Study	Zhao et al. (52)	2022	China	Hospital-based randomized controlled trial	560 women (280 vs. 280)	>18 y of age, singleton pregnancy, normoglycemic, overall healthy	MD advice with supplemented olive oil (40 mL/d) and pistachios (25–30 g/d); recommended 30 min/d of walking	Standard dietary guideline with limited fat intake; received lifestyle guideline including PA	Reduced gestational hypertension (RR 0.67; 95% CI, 0.24–1.85)	Non-significant reduction in gestational hypertension	Strengths: Randomized design, early intervention, provision of olive oil and nuts Limitations: Adherence to intervention not biochemically confirmed
12. PREDG Study	Barquiel et al. (53)	2023	Spain	Randomized controlled trial	169 women	Women with pregestational obesity (BMI ≥ 30 kg/m^2^)	Group education program on Mediterranean diet (50% carbohydrates, 20% protein, 30% fat) and physical activity	Conventional follow-up with standard dietary advice	Gestational weight gain; pregnancy complications (gestational diabetes, hypertension/preeclampsia)	Reduced rates of hypertension/preeclampsia (18.2% vs. 32.5%, p = 0.040)	Strengths: Randomized design, focus on high-risk obese population Limitations: Small sample size, combined intervention with physical activity

MD, Mediterranean diet; EVOO, Extra Virgin Olive Oil; HDP, Hypertensive Disorders of Pregnancy; GDM, Gestational Diabetes Mellitus; SGA, Small for Gestational Age; GW, Gestational Week; PA, Physical Activity; MDA, Mediterranean Diet Adherence; AMED, Alternate Mediterranean Diet; MSDS, Mediterranean-Style Diet Score; aMed, Alternate Mediterranean Diet; RCT, Randomized Controlled Trial; RR, Relative Risk; OR, Odds Ratio; aOR, Adjusted Odds Ratio; CI, Confidence Interval; BMI, Body Mass Index; APO, Adverse Pregnancy Outcome.

## 4 Discussion

### 4.1 Overview of the findings

This literature review aimed to synthesize current evidence on the Mediterranean diet (MD) and preeclampsia prevention by analyzing both mechanistic pathways and clinical evidence. Our analysis of twelve studies, comprising five observational studies and seven randomized controlled trials (RCTs), suggests that higher adherence to the Mediterranean diet is associated with a reduced risk of preeclampsia and other hypertensive disorders of pregnancy. The protective effect appears consistent across different study designs, populations, and outcome definitions, reinforcing the robustness of these findings.

### 4.2 Analysis of observational studies

The observational studies in our review demonstrated substantial protective associations between Mediterranean diet adherence and preeclampsia risk. The Australian Longitudinal Study on Women’s Health (42) reported a 42% lower risk of developing hypertensive disorders during pregnancy among women in the highest quartile of Mediterranean diet adherence compared to those in the lowest quartile (RR 0.58; 95% CI, 0.42–0.81). This large, population-based prospective cohort provides valuable evidence on the potential protective effects of pre-pregnancy dietary patterns on hypertensive disorders in pregnancy risk.

Similarly, the Boston Birth Cohort (45) conducted in a racially diverse, urban, low-income population found that higher Mediterranean diet adherence was associated with lower preeclampsia odds (highest vs. lowest tertile aOR 0.78, 95% CI 0.64–0.96), with stronger protective effects observed among Black women. This finding is particularly important as it suggests the benefits of Mediterranean diet may extend across different racial and socioeconomic groups, potentially addressing disparities in preeclampsia risk (54, 55).

The nuMoM2b Study (46) further strengthened this evidence, reporting a 28% reduction in preeclampsia odds (aOR 0.72, 95% CI 0.55–0.93) with high versus low aMed score based on periconceptional dietary intake. The dose–response relationship observed in this large multicenter cohort provides additional support for a causal relationship between Mediterranean diet adherence and preeclampsia risk (56).

A notable feature of these observational studies is their methodological rigor, including prospective designs, adequate sample sizes, and comprehensive adjustment for potential confounders such as age, BMI, physical activity, and medical history. This strengthens the validity of their findings despite the inherent limitations of observational research (5).

### 4.3 Analysis of interventional studies

The RCTs in our review showed promising, albeit mixed, results regarding Mediterranean diet interventions and preeclampsia risk. The ESTEEM trial (48), which randomized 1,138 pregnant women with metabolic risk factors to a Mediterranean diet intervention supplemented with mixed nuts and extra virgin olive oil (EVOO) or standard care, reported a non-significant trend toward reduction in preeclampsia (aOR 0.76, 95% CI 0.56–1.03). Despite not reaching statistical significance, the magnitude and direction of the effect align with the observational evidence.

Similarly, the study by Crovetto et al. (51) found a reduced risk of preeclampsia in the Mediterranean diet group (RR 0.61; 95% CI, 0.37–1.01) that approached statistical significance. This trial’s strengths include its large sample size (n = 1,184) and multi-arm design, allowing comparison of Mediterranean diet with other interventions such as mindfulness-based stress reduction.

The interventional approach used in these trials typically involved dietary advice on Mediterranean diet principles, often supplemented with key Mediterranean diet components such as EVOO (1–2 L/month) and nuts (15–30 g/day). This approach is practical for clinical implementation while ensuring adequate exposure to the bioactive components hypothesized to mediate Mediterranean diet’s beneficial effects (57, 58).

Interestingly, several RCTs showed stronger effects on related outcomes such as gestational diabetes mellitus and preterm birth. For instance, the St. Carlos GDM Prevention Study (47) demonstrated significant reductions in pregnancy-induced hypertension with a Mediterranean diet intervention supplemented with EVOO and pistachios, highlighting the potential broader benefits of Mediterranean diet for pregnancy complications sharing common pathophysiological pathways with preeclampsia (14, 59).

### 4.4 Mechanisms linking Mediterranean diet to preeclampsia prevention

Several biological mechanisms likely underlie the protective effects of the Mediterranean diet against preeclampsia, addressing key pathophysiological processes involved in this disorder:

#### 4.4.1 Anti-inflammatory effects

Preeclampsia is characterized by excessive systemic inflammation, with elevated levels of pro-inflammatory cytokines and immune cell activation (60, 61). The Mediterranean diet ‘s anti-inflammatory properties derive from its high content of polyphenols, omega-3 fatty acids, and other bioactive compounds found in fruits, vegetables, olive oil, and nuts (62, 63) These components modulate inflammatory pathways by inhibiting NF-κB activation, reducing pro-inflammatory cytokine production, and enhancing anti-inflammatory mediators (64, 65).

Clinical trials have demonstrated that Mediterranean diet interventions reduce systemic inflammatory markers such as C-reactive protein, interleukin-6, and tumor necrosis factor-alpha (33, 66). In pregnancy specifically, Mikkelsen et al. (67) found that greater adherence to Mediterranean diet-like dietary patterns was associated with lower levels of inflammatory markers in maternal circulation. This anti-inflammatory effect may help mitigate the exaggerated inflammatory response characteristic of preeclampsia.

#### 4.4.2 Antioxidant properties

Oxidative stress plays a central role in preeclampsia pathophysiology, contributing to placental dysfunction, impaired trophoblast invasion, and systemic endothelial damage (68, 69). The Mediterranean diet is abundant in antioxidants, including vitamins C and E, carotenoids, flavonoids, and polyphenols from fruits, vegetables, olive oil, and nuts (22, 70).

These antioxidants neutralize reactive oxygen species, reduce lipid peroxidation, and enhance endogenous antioxidant defenses (71). A randomized trial by Hernáez et al. demonstrated that Mediterranean diet supplemented with EVOO significantly improved markers of oxidative stress and enhanced antioxidant capacity (72). In pregnancy, improved antioxidant status could protect against placental oxidative damage and subsequent preeclampsia development (73).

#### 4.4.3 Vascular and endothelial function

Endothelial dysfunction is a hallmark of preeclampsia, manifesting as increased vascular reactivity, impaired vasodilation, and enhanced platelet aggregation (74). Several components of the Mediterranean diet, particularly EVOO, nuts, and fish, improve endothelial function (75, 76).

Olive oil polyphenols enhance nitric oxide production and bioavailability, promoting vasodilation and reducing blood pressure (32). Omega-3 fatty acids from fish modulate endothelial cell function, reduce vasoconstrictor production, and improve vascular compliance (77). Clinical trials have demonstrated that Mediterranean diet interventions improve flow-mediated dilation, reduce blood pressure, and enhance endothelial-dependent vasodilation (78, 79).

These vascular benefits may be particularly important in preeclampsia prevention, as they directly counteract the vascular dysfunction underlying its clinical manifestations (80, 81).

#### 4.4.4 Angiogenic balance

An imbalance between pro-angiogenic factors (PlGF) and anti-angiogenic factors (sFlt-1, sEng) is central to preeclampsia pathophysiology (82, 83). Evidence suggests that nutritional factors can influence this balance, with potential implications for preeclampsia prevention (35).

The omega-3 fatty acids in fish, a key component of the Mediterranean diet, have been shown to promote pro-angiogenic factor expression while reducing anti-angiogenic factor production (84). Polyphenols from fruits, vegetables, and olive oil also modulate angiogenic pathways, potentially improving placental vascularization (85). In a study by Garcia-Aloy et al., consumption of Mediterranean diet components was associated with altered levels of angiogenesis biomarkers in a direction consistent with improved vascular health (86).

#### 4.4.5 Metabolic effects

Metabolic disturbances, including insulin resistance, dyslipidemia, and obesity, increase preeclampsia risk and contribute to its pathogenesis (87, 88). The Mediterranean diet improves insulin sensitivity, optimizes lipid profiles, and supports healthy weight management (89, 90).

Whole grains, legumes, and vegetables in the Mediterranean diet provide complex carbohydrates and fiber that regulate glucose metabolism and reduce insulin resistance (91). The healthy fats in olive oil and nuts improve lipid profiles and reduce atherogenic risk (92). These metabolic benefits may be particularly relevant for preeclampsia prevention, given the overlapping pathophysiology between metabolic syndrome and preeclampsia (27, 93).

#### 4.4.6 Gut microbiota

Emerging evidence suggests that gut dysbiosis may contribute to preeclampsia pathogenesis through increased bacterial translocation, endotoxemia, and systemic inflammation (34, 94). The high fiber content of the Mediterranean diet from fruits, vegetables, whole grains, and legumes promotes a healthier gut microbiome (95, 96). However, this microbiota transformation is not immediate, as the replacement of deleterious gut bacteria with beneficial strains requires time to establish stable colonization and metabolic activity. The NU-AGE study demonstrated that meaningful alterations in gut microbiome composition following Mediterranean diet intervention occur over extended periods, with significant changes observed after 1 year of sustained dietary adherence (96).

**Figure 1:**
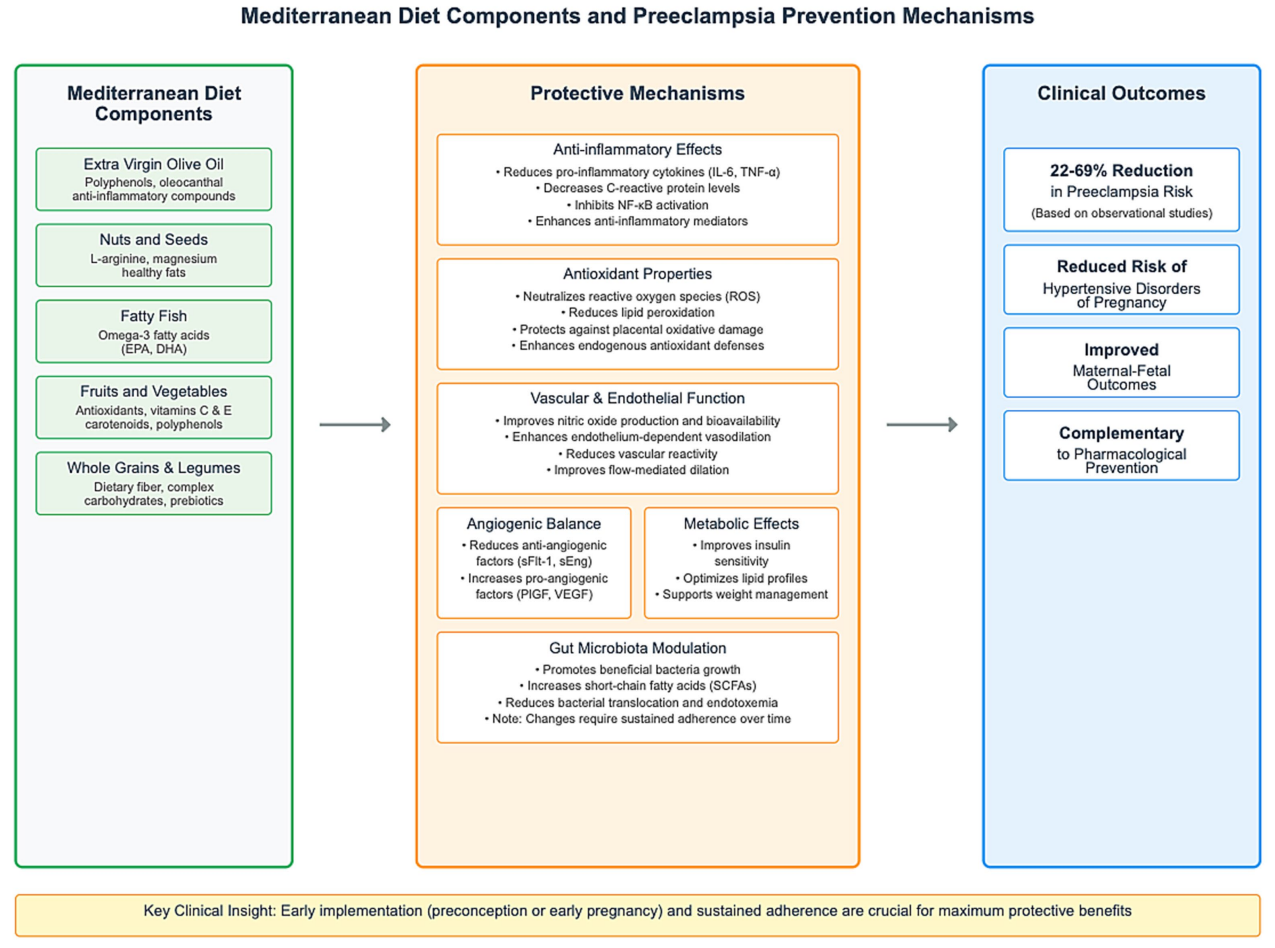
Mediterranean diet and preeclampsia prevention mechanisms.

These dietary fibers serve as prebiotics, fostering the growth of beneficial bacteria that produce short-chain fatty acids (SCFAs) with anti-inflammatory and barrier-enhancing properties (28). The temporal nature of this microbial shift underscores the importance of early and sustained dietary intervention, as the De Filippis study demonstrated that high-level adherence to Mediterranean diet patterns was associated with increased fecal concentrations of SCFAs and beneficial microbiota composition (28). Additional research has confirmed that shifts in gut microbiota associated with Mediterranean diet adherence require sustained dietary modifications to achieve optimal microbial diversity and metabolic benefits (97, 98). This delayed but sustained microbiome modulation represents an additional mechanism by which the Mediterranean diet may protect against preeclampsia, though the protective effects may be most pronounced when dietary changes are implemented well before conception or early in pregnancy to allow sufficient time for beneficial microbial establishment and stabilization (Figure 1).

### 4.5 Timing of Mediterranean diet for maximum benefit

A critical aspect of optimizing Mediterranean diet interventions for preeclampsia prevention is determining the ideal timing for implementation. Our analysis suggests that the protective effects may vary depending on when the Mediterranean diet is adopted relative to conception and pregnancy stages.

The observational studies by Schoenaker et al. (42) and Makarem et al. (46) demonstrated significant protective associations between preconception Mediterranean diet adherence and subsequent preeclampsia risk. This aligns with our understanding that preeclampsia pathogenesis begins with abnormal placentation in early pregnancy, which may be influenced by preconception nutritional status (29, 99).

The interventional studies in our review initiated Mediterranean diet interventions at various gestational ages, ranging from 8 to 12 weeks (47, 50) to 19–23 weeks (51). Interestingly, some evidence suggests greater benefits with earlier intervention. For example, in the meta-analysis by Yang *et al*., effect sizes for preterm birth were greater in RCTs that initiated interventions in the first trimester versus later (54).

This timing consideration has important implications for clinical implementation. Ideally, Mediterranean diet promotion should begin preconceptionally and continue through early pregnancy to maximize potential benefits (100, 101). This approach would address both the early placentation processes and the subsequent maternal systemic response that characterizes preeclampsia (102).

### 4.6 Potential applications in clinical practice

Despite limitations in the current evidence base, the available data support considering the Mediterranean diet as a promising dietary approach for preeclampsia prevention, particularly among high-risk women. Several potential clinical applications warrant consideration. Women at elevated preeclampsia risk, including those with previous preeclampsia, chronic hypertension, obesity, or autoimmune conditions, could be specifically targeted for Mediterranean diet interventions (103, 104), aligning with current trends in precision nutrition and personalized medicine (105). The Mediterranean diet could complement existing preeclampsia prevention strategies, including low-dose aspirin and calcium supplementation in indicated populations (106, 107), potentially providing additive benefits through complementary mechanisms of action (108). Successfully implementing Mediterranean diet interventions requires addressing practical barriers to adherence through Mediterranean diet cooking classes, meal planning resources, grocery shopping guides, and strategies for adapting Mediterranean diet principles to various cultural and socioeconomic contexts (107, 109). Incorporating Mediterranean diet recommendations into preconception care programs could optimize nutritional status before pregnancy, potentially improving early placentation and reducing preeclampsia risk (110), aligning with growing recognition of preconception health’s importance for optimal pregnancy outcomes (111).

### 4.7 Limitations of current evidence

Several critical limitations affect evidence interpretation. Mediterranean diet adherence assessment varies considerably across studies, with different scoring systems showing only 65% agreement rates, complicating direct comparisons (112–114). Most research has been conducted in predominantly White populations, raising generalizability concerns given preeclampsia’s disproportionate impact on racial and ethnic minorities (115–117). Interventional approaches varied significantly across RCTs in terms of dietary advice, supplementation protocols, and follow-up intensity (118). While most studies adjusted for important confounders, residual confounding by unmeasured factors and inconsistent evaluation of effect modifiers remain concerns (119, 120).

### 4.8 Future research directions

Priority research areas should address current evidence gaps to optimize clinical implementation. Standardized, validated methods for assessing Mediterranean diet adherence are essential, with objective biomarkers such as plasma fatty acid profiles or urinary polyphenol metabolites complementing self-reported dietary data (121, 122). Research in diverse populations is crucial, particularly among groups with higher preeclampsia risk such as Black and South Asian women, examining whether protective effects vary by race/ethnicity and how cultural adaptations influence outcomes (123–126).

Mechanistic studies examining specific pathways implicated in preeclampsia development would enhance understanding, with measurements of inflammatory markers, angiogenic factors, and endothelial function providing valuable insights (30, 40). Investigation of optimal timing, duration, and specific Mediterranean diet components is needed, exploring whether particular elements contribute disproportionately to protection (127). Finally, research addressing barriers and facilitators to Mediterranean diet adherence among pregnant women from diverse backgrounds, including consideration of cultural preferences, socioeconomic factors, and food access, is essential for successful implementation (128, 129).

## 5 Conclusion

This comprehensive review demonstrates that Mediterranean diet adherence is associated with reduced preeclampsia risk through multiple complementary mechanisms, including anti-inflammatory effects, improved endothelial function, and beneficial microbiota modulation. Observational studies consistently show 22–69% risk reductions, while interventional trials demonstrate promising trends toward protection.

The Mediterranean diet represents a safe, accessible, non-pharmacological approach to preeclampsia prevention with established broader health benefits. Priority research directions include developing standardized adherence assessment methods, expanding studies to diverse populations, and investigating optimal intervention timing and components. Implementation strategies should focus on early dietary counseling, cultural adaptation, and integration with existing prevention protocols.

Based on current evidence, Mediterranean diet promotion merits consideration in clinical practice, particularly for women at elevated preeclampsia risk, while acknowledging the need for continued research to optimize intervention strategies and confirm benefits across diverse populations.
